# Supplementary material for: Targeted Mutagenesis of the Multicopy Myrosinase Gene Family in Allotetraploid Brassica juncea Reduces Pungency in Fresh Leaves across Environments
Source: Plants (Basel). 2022 Sep 23;11(19):2494. doi: 10.3390/plants11192494 (PMC9572489; doi:10.3390/plants11192494)
Supplement: Supplementary file 1 [file plants-11-02494-s001.zip › plants-1888607-supplementary.pdf]

Table S1. Plasmid purity test results

**Sample**	**Red Giant edited line**	**MPS798_S43 (unedited negative control 1)**	**MPS799_S44 (unedited negative control 2)**	**CE73908_S50 (Transgene positive)**	**MPS801_S30 (1:5 dilution of Transgene positive)**
raw reads	264024	543992	588749	509747	**377808**
quality reads	263871	542053	587151	508896	**375882**
mapped reads	262698	518478	524618	504231	**354495**
quality read percent	99.9	99.6	99.7	99.8	**99.5**
mapped read percent	99.4	95.6	89.3	99	**94.2**
mapped reads *B. juncea* control gene1	11913	16999	22439	17673	**8917**
mapped reads *B. juncea* control gene2	11051	18789	21761	18174	**14273**
mapped reads *B. juncea* control gene1 %	4.53	3.28	4.28	3.5	**2.52**
mapped reads *B. juncea* control gene2 %	4.21	3.62	4.15	3.6	**4.03**
mapped reads pWISE total	68	211	262	156533	**117921**
mapped reads plasmid total percent	0.03	0.04	0.05	31.04	**33.26**
Result	negative	negative	negative	positive	**positive**

Table S2. Sensory data summary demonstrating non-pungent perception of edited Red Giant material with multiple tasters

	**Average**	**Std. Dev.**	**n samples**	**n tasters**
**edited RG**	**1.0**	**0.0**	**30**	**7**
**unedited RG**	**3.7**	**0.95**	**7**	**4**

Note: non-pungent (score of 1), slightly pungent (score of 2), moderately pungent (score of 3), pungent (score of 4) and very pungent (score of 5)

Table S3. ANOVA table for reduced model with factors for the cultivar, harvest stage, environment and interaction effect harvest stage and environment. The interaction and cultivar terms were highly significant factors for the GRA levels at an alpha level of 0.5

**Effect**	DF	Sum of Squares	Mean Square	F-Value	p-value
Cultivar	1	50.318	50.318	278.78	<0.0001
Harvest Stage	1	0.003	0.003	0.02	0.9
Environment	2	0.466	0.233	1.28	0.2873
Harvest Stage: Environment	2	6.524	3.262	17.88	<0.0001
Residuals	52	9.488	0.183		

Table S4. Post-hoc pairwise comparisons for glucose release assay absorption as a proxy for myrosinase activity between unedited and edited Red Giant at different harvest stages and between environments derived from the analysis of variance model (ANOVA) with factors for the cultivar, harvest stage, environment and associated interactions. At a significance level of 0.5, the unedited Red Giant line had significantly larger GRA estimates than the edited Red Giant line across environments and harvest stages. Estimated differences between the edited Red Giant and unedited Red Giant lines are the same across harvests stages and environments because the environment by harvest stage interaction term is not a factor for within environment and harvest stage comparisons.

Line #1	Line #2	Environment	Harvest Stage	Estimated Mean GRA Difference	Standard Error	DF	T-Statistic	p-value
Unedited Red Giant	Edited Red Giant	Growth Chamber, Durham, NC	Baby	1.84	0.11	52	16.34	<0.0001
Unedited Red Giant	Edited Red Giant	Salinas, CA	Baby	1.84	0.11	52	16.34	<0.0001
Unedited Red Giant	Edited Red Giant	Yuma, AZ	Baby	1.84	0.11	52	16.34	<0.0001
Unedited Red Giant	Edited Red Giant	Growth Chamber, Durham, NC	Mature	1.84	0.11	52	16.34	<0.0001
Unedited Red Giant	Edited Red Giant	Salinas, CA	Mature	1.84	0.11	52	16.34	<0.0001
Unedited Red Giant	Edited Red Giant	Yuma, AZ	Mature	1.84	0.11	52	16.34	<0.0001

Table S5. Monthly maximum, average, and minimum temperature and humidity, and monthly precipitation for Moses Lake, WA during the 2021 growing season.

	Temperature (°C)			Humidity (%)			Precipitation (mm)
Month	Max	Avg	Min	Max	Avg	Min	Total
May	32.2	17.5	3.3	63.6	36.6	18.1	2
June	45.6	24.5	7.8	59.6	35.0	16.6	5
July	41.7	26.7	11.1	63.4	34.9	15.4	0
August	38.3	23.0	7.8	69.7	42.6	22.5	4

Table S6. Monthly maximum, average, and minimum temperature and humidity, and monthly precipitation for Yuma, AZ during the 2021/2022 growing season.

	Temperature (°C)			Humidity (%)			Precipitation (mm)
Month	Max	Avg	Min	Max	Avg	Min	Total
November	32.8	21.2	10.0	63.6	36.6	18.1	0
December	29.4	14.9	5.0	59.6	35.0	16.6	6
January	24.4	14.7	3.3	63.4	34.9	15.4	1
February	30.0	15.9	2.2	69.7	42.6	22.5	1

Table S7. List of primers used for pWISE687 and edit characterization.

Primer name	sequence	Direction	Purpose
NGS1PM1723	GACCGCCAGATCATGTATGT	Forward	NGS assay primer
NGS1PM1724	GACCGCCAGATCATGTAGGT	Forward	NGS assay primer
NGS1PM1725	GACCCTCAGATCATGTATGA	Forward	NGS assay primer
NGS1PM1726	GACCCCCAGATCATGTATGT	Forward	NGS assay primer
NGS1PM1727	GATCTTCCCTCCTTGGAACTG	Reverse	NGS assay primer
NGS1PM1728	AATCTTCCCTTGTTGGTCCTG	Reverse	NGS assay primer
NGS1PM1729	AATCTTCCCTTTTTGGAACTGT	Reverse	NGS assay primer
NGS1PM1730	AATCTTCCCGTTTTGGAACTG	Reverse	NGS assay primer
NGS1PM1731	AATCTTCCCTTTTTGAAACTGTT	Reverse	NGS assay primer
NGS1PM1732	AATCTTCCCTTTTTGGAACTGC	Reverse	NGS assay primer
NGS1PM1733	AATCTTACCTCCTTGATGCTGT	Reverse	NGS assay primer
TQ144	AGTACTGTTCGTGGGCGAGAGA	Forward	qPCR assay
TQ149	TCCCCGACGGACGTAAAG	Reverse	qPCR assay
TQ151	TTACGCATCCATGACTG	MGB probe	qPCR assay
TQ00135	CATCGAAACTATCCTCCCAGAGTT	Forward	qPCR assay
TQ00136	GCGGTTGTGAACCCGTTAAA	Reverse	qPCR assay
TQ0117	TCGCGCTGGTGAAC	MGB probe	qPCR assay
TQ0065	TATGGACATTTTCGAGAAGGTGG	Forward	qPCR assay
TQ0066	CGACGTCATAATCGGAATTGAGA	Reverse	qPCR assay
TQ0043	CGATCTTCGATAAGCAC	MGB probe	qPCR assay
TQ0124	TGCCTGACATCCGCCTACA	Forward	qPCR assay
TQ0125	AGGGCCATGAAGCTGGAGTAA	Reverse	qPCR assay
TQ0126	CTGTGAGCAGTCCGAC	MGB probe	qPCR assay
NGS1PM3801	ACCACCAACTCATAGATGCAC	Forward	1.3 left + 1.4 right
NGS1PM3802	ACCATTTAATGTAAACAAACAA	Reverse	1.3 left + 1.4 right
NGS1PM2729	TATAGTACTTACCTGGGAGCT	Forward	1.4 left + 1.5 right
NGS1PM3731	TCTAGTTATACAGACCACCTTTAG	Reverse	1.4 left + 1.5 right

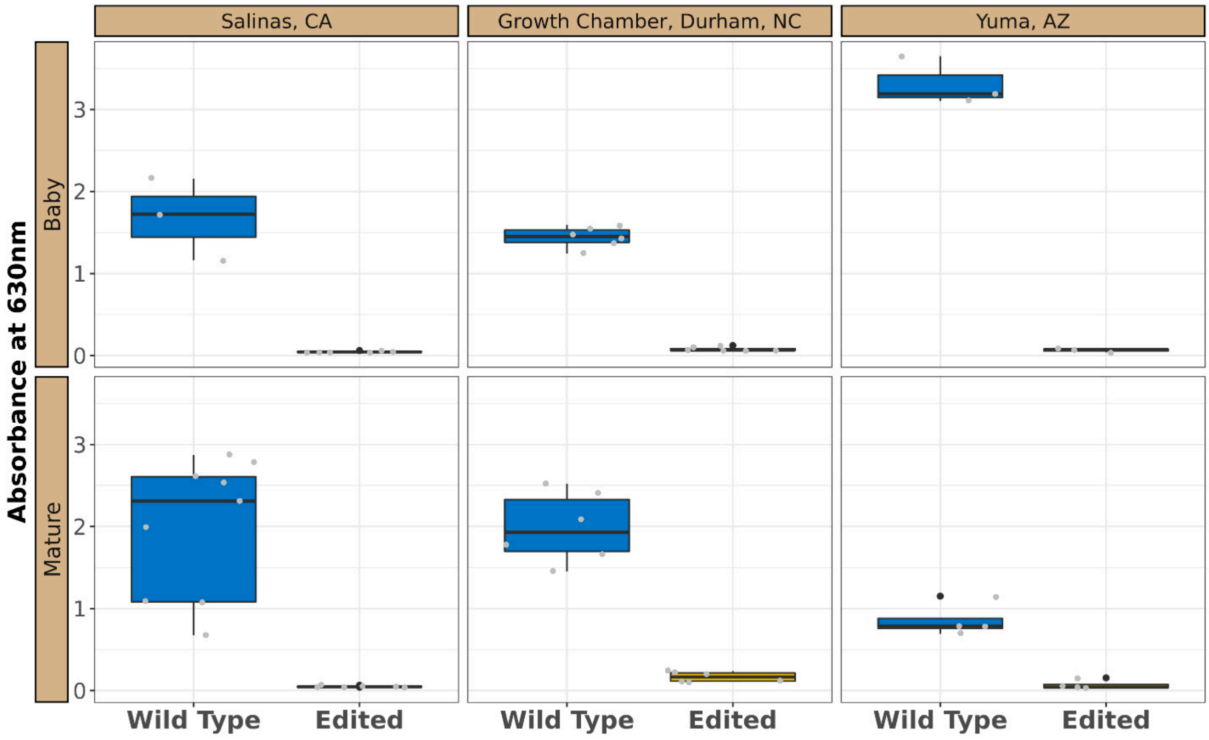

Figure S1. Distribution of glucose release absorbance values at 630 nm from leaf discs in the presence sinigrin for unedited and edited Red Giant mustard cultivars by environment and harvest stage. Leaf samples were randomly selected from harvested material. Baby leaves were harvested 31 days post wet date in Salinas, CA on 9/20/2021; 19 days post wet date in the growth chamber on 1/4/2022; and 46 days post wet date in Yuma, AZ on 1/4/2022. Mature leaves were harvested 61 days post wet date in Salinas, CA on 12/2/2021; 48 days post wet date in the growth chamber on 12/16/2021; and 75 days post wet date in Yuma, AZ on 2/2/2022. Data values are the GRA colorimetric absorbance values at 630 nm from leaf discs processed in the presence of sinigrin.

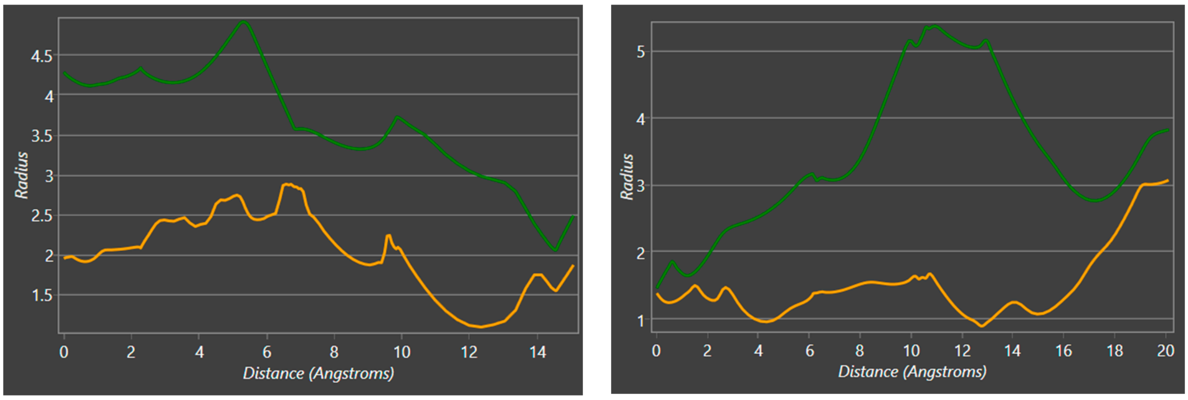

(A) (B)

Figure S2: Radius (orange) and free radius (green) measurements of pockets identified in wild-type (A) and edited (B) B05 myrosinase using MOLE2.5. Radius measurements indicate the radius of the largest possible sphere that could fit at a given point along the pocket, while free radius measurements indicate the distance from the center of the pocket to the protein backbone and do not account for side chain identities or conformations.

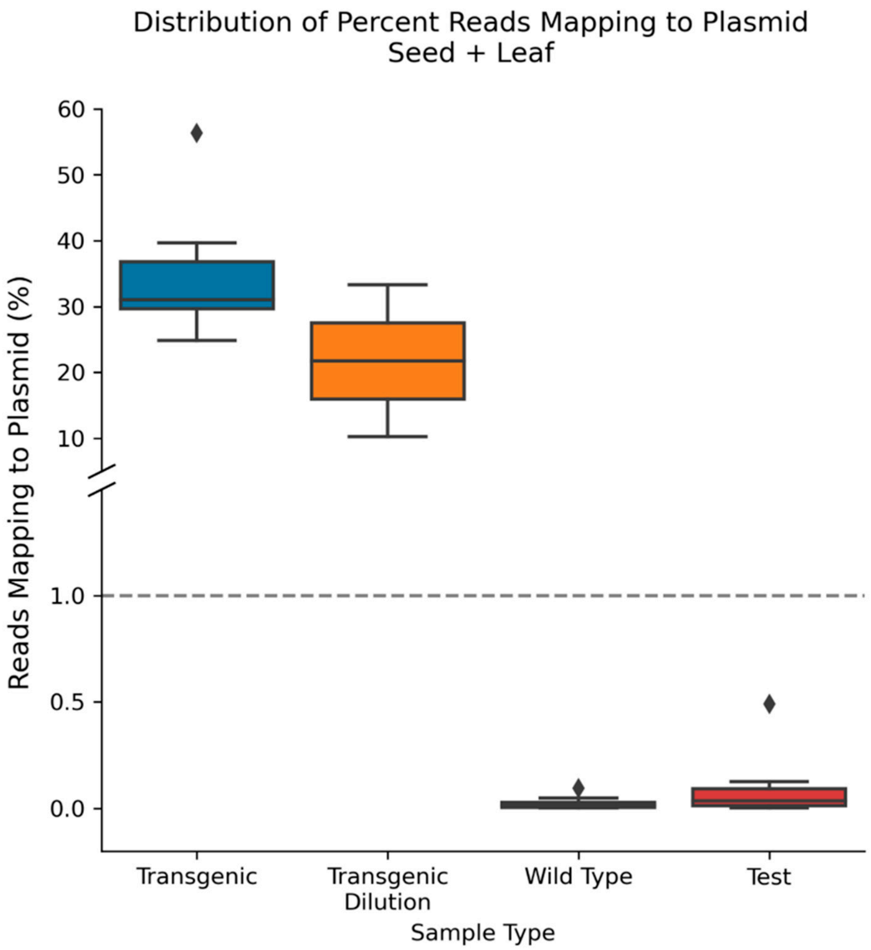

Figure S3. Standard boxplots showing distribution of % reads mapping to the plasmid in transgenic and control samples. The gray line indicates the 1% threshold.

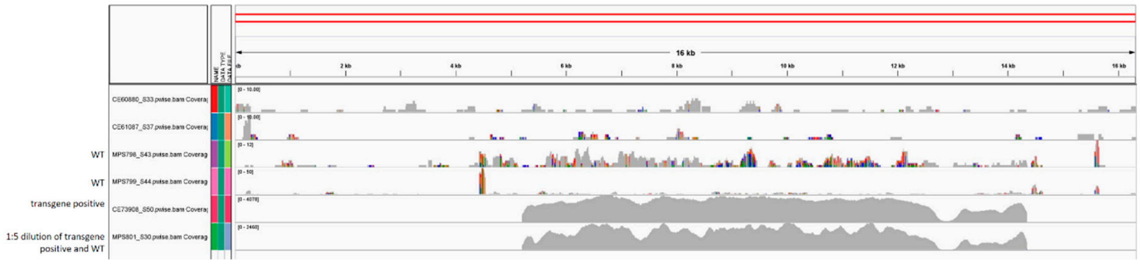

Figure S4. IGV screenshot showing read alignment histograms to the plasmid sequence for edited lines (coverage$_{max}$ = 10x, 10x), two wild-type samples (coverage$_{max}$ = 12x, 50x), transgene positive sample (coverage$_{max}$ = 4,078x), and 1:5 dilution of transgene positive sample in WT (coverage$_{max}$ = 2,460x). Note that y-axis scales for read coverage are variable and labeled on the left side of each track.

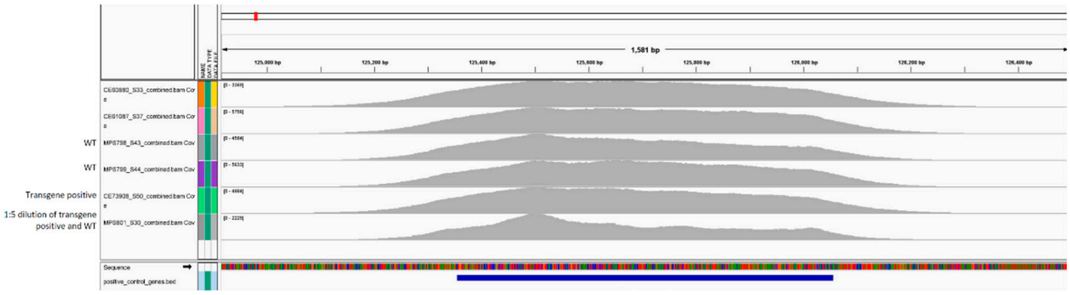

Figure S5. IGV screenshot showing read alignment histograms to positive control gene 1 sequence for edited lines (coverage$_{max}$ = 3,369x, 5,756x), two unedited samples (coverage$_{max}$ = 4,584x, 5,633x), transgene positive sample (coverage$_{max}$ = 4,984x), and 1:5 dilution of transgene positive sample in unedited *B. juncea* (coverage$_{max}$ = 2,229x).

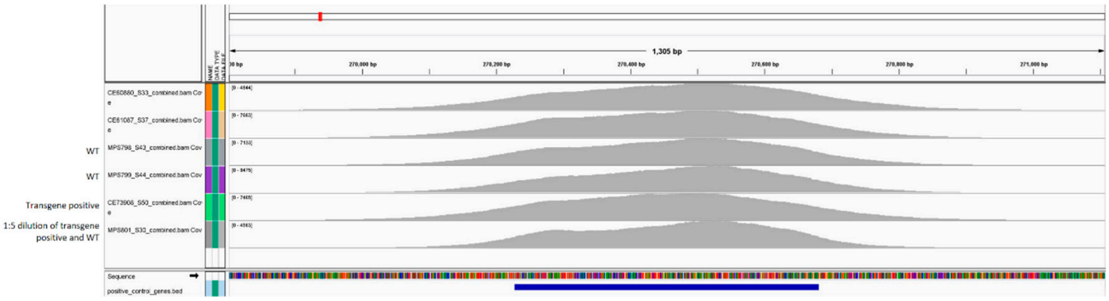

Figure S6. IGV screenshot showing read alignment histograms to positive control gene 2 sequence for edited lines ($coverage_{max}$ = 4,844x, 7,633x), two unedited samples ($coverage_{max}$ = 7,133x, 8,475x), transgene positive sample ($coverage_{max}$ = 7,465x), and 1:5 dilution of transgene positive sample in unedited *B. juncea* ($coverage_{max}$ = 4,563x).
